# Vehicle speed measurement method using monocular cameras

**DOI:** 10.1038/s41598-025-87077-6

**Published:** 2025-01-22

**Authors:** Hao Lian, Meian Li, Ting Li, Yongan Zhang, Yanyu Shi, Yikun Fan, Wenqian Yang, Huilin Jiang, Peng Zhou, Haibo Wu

**Affiliations:** 1 Computer and Information Engineering College, Inner Mongolia Agricultural University, Hohhot, 010000 China; 2 Inner Mongolia Autonomous Region Key Laboratory of Big Data Research and Application of Agriculture and Animal Husbandry, Hohhot, China

**Keywords:** Monocular vision, Two-dimensional positioning, Camera calibration, YOLOv7, DeepSORT, Computer science, Imaging techniques

## Abstract

This paper proposes a method for fast and accurate vehicle speed measurement based on a monocular camera. Firstly, by establishing a new camera imaging model, the calibration method for variable focal lengths is optimized, simplifying the transformation process between the four coordinate systems in traditional camera imaging models, and the method does not need to restore the pixel coordinates to dedistortion. Secondly, based on the camera imaging model, a two-dimensional positioning algorithm is proposed. By leveraging the characteristics of the speed measurement problem, the complex three-dimensional positioning problem is simplified into a two-dimensional model, reducing the overall computational complexity of the positioning problem. Finally, the algorithm is combined with You Only Look Once version 7 (YOLOv7) and Deep Simple Online and Realtime Tracking (DeepSORT) algorithms, integrating multiple model structures to optimize the network, achieving precise multi-target speed measurement. Experiments show that under frame-by-frame measurement conditions, the minimum and average accuracies of this method reach 95.1% and 97.6%, respectively. Compared with other methods, it has significant advantages in speed measurement accuracy and computational efficiency. Therefore, this research outcome is expected to play an important role in intelligent transportation systems and road safety management.

## Introduction

According to statistics^1^, there were 233,709 traffic accidents caused by motor vehicles in China in 2021, resulting in 294,284 casualties and direct economic losses of 134.5486 million yuan. According to the detailed original data on traffic accidents in Kunming, Yunnan Province collected by Hu Liwei et al.^2^, speeding was the main cause of 31.56% of traffic accidents, the highest among all factors. Therefore, how to quickly and accurately estimate vehicle speed has become one of the key directions in current intelligent transportation system research, which requires solving problems of target detection, target tracking, target positioning, and speed estimation simultaneously^3^.

Currently, vehicle speed measurement systems are mainly divided into intrusive sensor systems and non-intrusive sensor systems^4^. Intrusive sensor systems usually require loop detectors to be installed on the ground. The installation process is relatively complex, and the detectors are prone to wear and damage, which may also cause damage to the road^5^. Non-intrusive sensor systems mainly use devices such as LiDAR^6^, magnetic sensors^7^, and cameras. Although they avoid the problems of intrusive sensors, they have strict installation conditions, are expensive, and have high maintenance costs. With the rapid development of camera technology and computer vision technology, using cameras to obtain data has gradually become one of the most effective mainstream solutions for non-intrusive traffic data collection^8^.

When using cameras for speed estimation, there are issues such as slow target detection speed, low accuracy of positioning algorithms, and high equipment purchase and maintenance costs. Vehicle speed measurement methods based on monocular cameras usually include several key tasks: target detection, multi-target tracking, three-dimensional positioning, and speed measurement. For target detection and tracking algorithms, deep learning-based target detection algorithms (such as You Only Look Once (YOLO), Retina Neural Network (RetinaNet), Fast Regions with Convolutional Neural Networks (Fast RCNN), etc.) have overcome the limitations of traditional algorithms in terms of real-time performance and accuracy and have been widely applied. The YOLO algorithm, as a representative of single-stage target detection algorithms, is one of the most effective solutions^9^. Additionally, combining multi-target tracking algorithms (such as Simple Online and Realtime Tracking (SORT),Deep Simple Online and Realtime Tracking (DeepSORT), Fair Multi-Object Tracking (FairMOT)) with target detection to enhance multi-target tracking performance is also a main research direction currently^10^.

The most important task in vehicle speed estimation is the transformation from images to the real world, in order to obtain the position information of objects in three-dimensional space from two-dimensional images. This paper proposes a camera imaging model that optimizes the calibration method for variable focal lengths, simplifies the transformation process between the four coordinate systems in traditional camera imaging models, and does not require undistortion of pixel coordinates. Then, based on this method, a two-dimensional positioning model is proposed, along with a compensation mechanism to improve positioning accuracy. Finally, considering the characteristics of the speed measurement problem, the complex three-dimensional positioning problem is simplified into a two-dimensional positioning problem.

In summary, the main contribution of this paper is the proposal of a method to estimate vehicle speed by accurately locating vehicle targets in video images captured by a monocular camera. This method has significant advantages in target positioning accuracy, overcoming the issue of decreased accuracy in measuring distant targets caused by the inverse square law^11^. Firstly, YOLOv7 + DeepSORT is used for target detection and tracking to extract the coordinates and motion trajectories of the measured targets in the video, and a two-dimensional positioning model based on a ranging model is used for target positioning and speed estimation. The performance of this method was also evaluated under different environmental conditions and compared with the latest related research results in the field, showing that the proposed method has excellent performance.

## Related work

The monocular camera rapid speed measurement system proposed in this paper covers research topics in camera calibration, target detection and tracking, target positioning, and speed estimation. This section will briefly introduce the related academic research on these topics.

### Research on target positioning and speed estimation

In the research of accurately estimating vehicle speed, determining the precise position of the vehicle in the world coordinate system is the basis for speed measurement. Under monocular camera conditions, current research categorizes target positioning and speed estimation methods into three types. The first type is based on virtual lines or areas^12^, which does not require complex camera calibration. It only requires drawing two lines on the road or simulating virtual lines and measuring their actual distance. When the vehicle passes the set lines, timing starts, and the vehicle speed is calculated based on the time taken to pass through this area. Due to the diversity of vehicle movements, such as turning, changing speed, stopping, and issues like perspective constraints and shadows, the results of this method are relatively unstable in practical applications.

The second type maps the three-dimensional space to the two-dimensional space through a linear transformation^13^. This method treats the image as a bird’s-eye view, allowing changes in pixel coordinates to be directly mapped to changes in world coordinates. This method involves obtaining the camera’s internal and external parameters and actual scene data, requiring high accuracy in camera calibration algorithms. This method involves obtaining the camera’s internal and external parameters and actual scene data, requiring high accuracy in camera calibration algorithms.

The third type of algorithm is based on distance estimation formulas^14^, requiring preset real data of the measured object, typically needing the preset dimensions of the vehicle or license plate. Because this algorithm relies on the real data of the measured object, it needs to be deployed in a vehicle classification system with a large amount of data. Additionally, due to the difficulty in accurately calculating the size of the license plate, the application conditions for this method are relatively stringent.

### Research on multi-target detection and tracking

Target detection is an essential technology and a popular research direction for separating the background from the object of interest in videos to extract target information. Traditional computer vision techniques usually use Haar-like features (Haar-like) classifiers and Adaptive Boosting (AdaBoost) machine learning algorithms for target detection^15^. In recent years, deep learning-based target detection algorithms have dominated vehicle detection. Regions with Convolutional Neural Networks (R-CNN) was the first to use deep models to extract image features, pioneering a new era of detection algorithms with an accuracy rate of 49.6%. Subsequently, excellent algorithms such as Single Shot MultiBox Detector (SSD)^16^, Faster Regions with Convolutional Neural Networks (Faster R-CNN)^17^, Mask Regions with Convolutional Neural Networks (Mask R-CNN)^18^, and YOLO^15^ have emerged in the field of deep learning, further improving the performance of target detection.

In the context of using deep learning for target detection, computer vision can now be applied to tracking moving vehicles. By enabling models to learn more features for individual vehicle identification, effective vehicle tracking can be achieved within urban areas^19,20^. Additionally, the combination of spatiotemporal vehicle trajectories and optimized visual feature recognition techniques can achieve large-scale tracking^21^.

### Research on camera calibration

In order to extract three-dimensional information from two-dimensional images, camera calibration plays a crucial role as a preliminary step for measuring the real world. The ability to obtain accurate real-world coordinates from pixel coordinates largely depends on the accuracy of camera calibration. In monocular camera calibration, the main goal is to construct the camera’s imaging model and clarify the geometric relationship between the real three-dimensional information of a certain position on the object’s surface and the corresponding image. There is a general expression for the camera calibration problem that describes the geometric relationship between the pixel points $(u,v)$ on the image plane and the real-world coordinates $({x}_{w},{y}_{w},{z}_{w})$:

1 $$\left[\begin{array}{c}{s}_{u}\\ {s}_{v}\\ s\end{array}\right]=\left[\begin{array}{ccc}{f}_{x}& 0& {c}_{x}\\ 0& {f}_{y}& {c}_{y}\\ 0& 0& 1\end{array}\right]\left[\begin{array}{cccc}{r}_{11}& {r}_{12}& {r}_{13}& {t}_{x}\\ {r}_{21}& {r}_{22}& {r}_{23}& {t}_{y}\\ {r}_{31}& {r}_{32}& {r}_{33}& {t}_{z}\end{array}\right]\left[\begin{array}{c}{x}_{w}\\ {y}_{w}\\ {z}_{w}\\ 1\end{array}\right]$$

Here, ${f}_{x}$ and ${f}_{y}$ are the focal lengths of the camera along the *X*-axis and *Y*-axis, respectively, and ${c}_{x}$ and ${c}_{y}$ are the image centers. These parameters are represented in matrix *K* as the internal parameters of the camera, while the rotation and translation parameters form a 3 × 3 rotation matrix *R* and a 3 × 1 translation vector *T*. Therefore, the monocular camera calibration problem can be seen as the estimation problem of *K*, *R*, and *T*^22^.

In summary, the field of computer vision has been conducting in-depth research on the aforementioned issues. In terms of target positioning and speed estimation, current methods still suffer from low accuracy or high computational resource consumption. Therefore, this study constructs a custom camera imaging model, combining the characteristics of speed measurement, simplifying the three-dimensional positioning problem into a two-dimensional positioning problem, achieving high accuracy with reduced computation time. In multi-target detection and tracking, machine learning algorithms have advantages in handling complex scenes and improving generalization capabilities. This paper improves the YOLOv7 model and uses it for target detection, combining it with the DeepSORT algorithm to achieve multi-target tracking. The main reasons are its excellent real-time performance and strong generalization capabilities. Additionally, YOLOv7 supports training multiple models to adapt to different specific scenarios, providing flexibility for diverse practical applications. For camera calibration, the goal is to extract accurate three-dimensional information from two-dimensional images to determine the relationship between pixel points in the image and real-world coordinates. Traditional calibration methods may not provide sufficient compensation for distortions caused by nonlinear imaging, leading to measurement inaccuracies in practical applications. The calibration method in this paper is mainly based on the methods proposed by Lixia Xue et al.^23^ and Tian Gao^24^, improving by compensating for distortions and defocusing phenomena caused by nonlinear imaging at each focal point, better adapting to experimental scenarios.

## Materials and methods

**Fig. 1:**
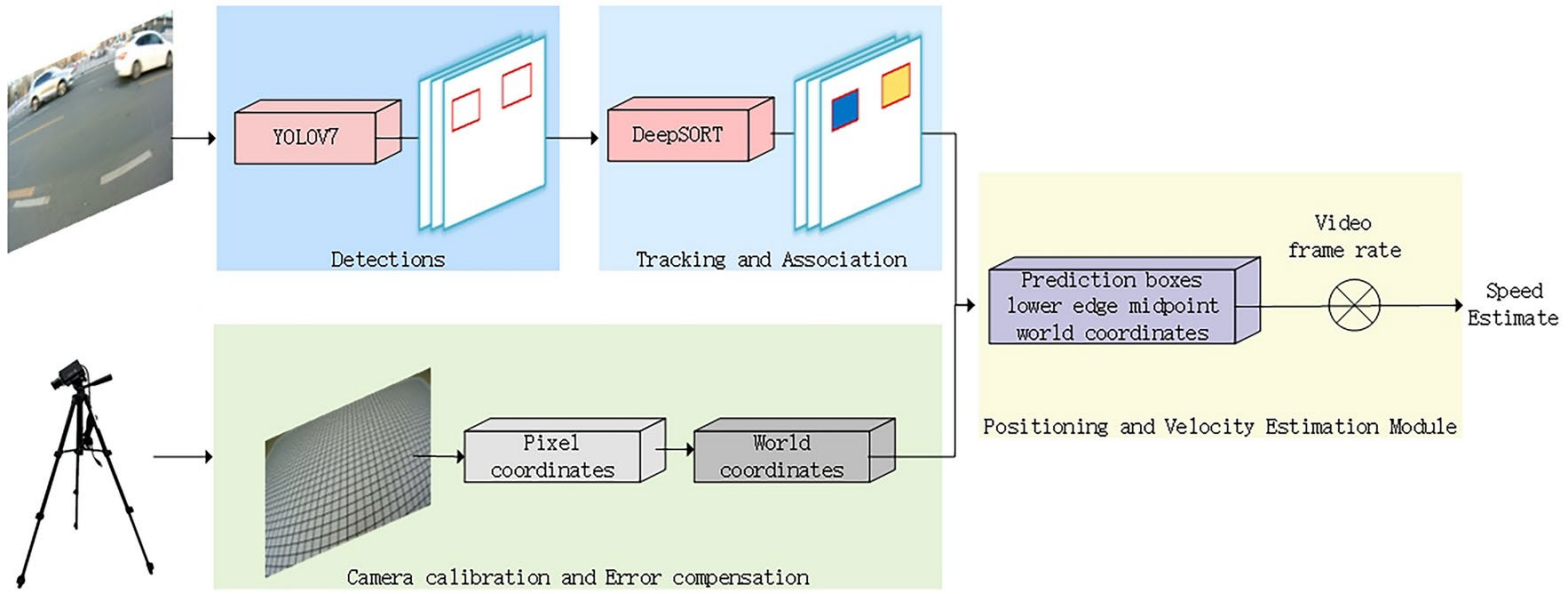
Overview of the vehicle speed estimation system.

This study achieves rapid measurement of the speed of the measured vehicle through video data. The process is mainly divided into four key stages: target detection and tracking, speed estimation, two-dimensional positioning, and camera calibration. First, the camera calibration process is performed by capturing images of a grid paper, extracting feature points on the grid paper for calibration. This process involves the conversion from pixel coordinates to world coordinates and the establishment of an error compensation mechanism to further optimize measurement accuracy. Subsequently, the captured vehicle video is input into the target detection network, where the YOLOv7 + DeepSORT algorithm is used to achieve multi-target tracking. Finally, by integrating the tracking results with the positioning algorithm, the pixel coordinates of the lower edge midpoint of the bounding box detected by the multi-target detection algorithm are extracted and converted to world coordinates for precise localization. The speed of the vehicle is then calculated based on the time difference between frames. The core contribution of this paper lies in proposing a monocular camera calibration method and positioning model, applying them to vehicle speed measurement. This approach not only reduces the complexity of traditional three-dimensional positioning but also improves speed measurement accuracy. This section will detail the proposed calibration method, positioning model, and their advantages when combined with speed measurement. The overall system overview is shown in Fig. 1:

### Target detection and tracking

#### Target detection

The performance of different detection algorithms was tested under the same conditions, and the comparison results are shown in Table 1. In the table, Parameters (Param) represents the number of parameters in the model, Floating Point Operations (FLOPs) denotes the number of floating-point operations required for each inference, Mean Average Precision from 50 to 95% IOU (mAP^val50–95^) indicates the mean average precision within the threshold range of 50% to 95%, and Frames Per Second (FPS) represents the number of frames that can be processed per second:

**Table 1:** Performance comparison of target detection algorithms.

Models	Param (M)	FLOPs (G)	mAP^val50–95^	FPS/frame∙s^−1^
YOLO v7	36.9	104.7	52.9	11.3
YOLO v8	68.2	257.8	53.9	9.1
YOLO v9	58.1	192.5	54.4	7.9
YOLO v10	29.5	160.4	54.4	10.4
YOLO v11	56.9	194.9	54.7	10.2

Under the same experimental conditions, YOLOv7 strikes a good balance between computational efficiency, resource usage, and accuracy due to its relatively low resource requirements and strong performance. This makes it more suitable for resource-constrained environments that require fast inference, meeting the demands of frame-by-frame processing. Therefore, YOLOv7 was selected as the target detection algorithm in this study. To ensure precise bounding boxes for small and medium-sized targets, the original multi-scale feature extraction network Panoptic Feature Pyramid Network (PaFPN) in YOLOv7 was replaced with Asymptotic Feature Pyramid Network (AFPN). AFPN initiates by integrating two adjacent low-level features and progressively incorporates high-level features. This approach avoids significant semantic gaps between non-adjacent levels and further employs adaptive spatial fusion operations to mitigate potential multi-target information conflicts during feature fusion. This modification allows YOLO to generate more reliable and tangent bounding boxes, accurately pinpointing object locations in the image.

In practical applications, the video is divided into frames, which are then fed into the YOLOv7 model for target detection. Upon obtaining the prediction boxes, the type of object and the pixel coordinates of the region containing the object are determined. The pixel coordinate information of the selected objects requiring measurement is then input into the positioning model for subsequent calculations.

#### Multi-target tracking

Target detection usually processes each frame of an image independently, without considering the continuity of the target between different frames. However, in actual vehicle speed measurement tasks, multi-target conditions are often required, necessitating the tracking of specific objects’ movements within a video sequence to associate the same objects across frames and track their positions. On the other hand, real road scenes often have a lot of occlusions, and the vehicles move quickly, demanding high performance from tracking algorithms. After comparing various target detection algorithms, this paper selects the DeepSORT algorithm to accomplish the multi-target tracking task. DeepSORT predicts the target’s motion trajectory using a Kalman filter^25^, whose core concept is to utilize the system’s dynamic model and observational data, recursively estimating the system state through prediction and update steps. For target matching and association, the Hungarian algorithm^26^ is applied to calculate similarity or distance between targets and detection boxes, effectively handling short-term occlusions and fast-moving targets, making DeepSORT highly suitable for vehicle speed measurement scenarios. Therefore, this paper chooses to combine the DeepSORT algorithm with YOLOv7 to achieve multi-target detection and tracking.

To optimize the tracking performance of the DeepSORT algorithm, this paper replaces the original feature library mechanism of DeepSORT with a feature update strategy. This strategy updates the appearance state of the i-th tracklet in the t-th frame using Exponential Moving Average (EMA), as shown in Eq. (2). Here, *x* is the feature of the object detected in the t-th frame assigned to the small tracklet *i*, and *e* is the feature of the small tracklet up to the t − 1 frame. By weighting with *α*, the feature is effectively updated, reducing noise.

2 $${e}_{i}^{t}=\alpha {e}_{i}^{t-1}+\left(1-\alpha \right){f}_{i}^{t}$$

The Noise Scale Adaptive Kalman algorithm (NSA Kalman)^27^ is introduced to replace the simple linear Kalman filter in DeepSORT, as assuming all detected objects have the same observation noise is unrealistic. The NSA Kalman filter adaptively changes the noise based on the detection confidence, allowing better tracking of complex objects. The improved multi-target tracking algorithm and its performance are shown in Table 2. The comparison results include the minimum accuracy, average accuracy, Multiple Object Tracking Accuracy (MOTA), Multiple Object Tracking Precision (MOTP), Identification F1 score (IDF1), Identification Precision (IDP), Identification Recall (IDR), and FPS of each method.

**Table 2:** Comparison of multi-target tracking algorithm performance.

Model	MOTA	MOTP	IDF1	IDP	IDR	FPS
YOLO + DeepSORT	74.8	75.3%	80.8%	81.0%	80.5%	10.6
Improved YOLO + DeepSORT	80.7%	82.9%	90.1%	91.0%	89.3%	9.8

### Speed estimation

Accurately estimating the speed of the vehicle being measured is the ultimate goal of this study. The motion of a vehicle is considered rigid body motion, so the principle of speed measurement is actually measuring the speed of any point on the vehicle. Typically, speed is measured through three-dimensional positioning. However, since the vehicle being measured is driving on a highway and the height of the vehicle’s feature points from the ground remains constant, the midpoint of the lower edge of the bounding box generated by the target detection algorithm can be considered as the vehicle’s coordinate. The speed of this midpoint can be regarded as the speed of the vehicle. Therefore, three-dimensional positioning can be simplified to two-dimensional positioning, and vehicle speed can be measured through two-dimensional positioning. After obtaining the pixel coordinates of the midpoint of the lower edge of the vehicle being measured, these coordinates are substituted into the two-dimensional positioning model to calculate the world coordinates of the object in the previous and subsequent frames. Then, the world coordinates are substituted into the Euclidean distance formula (3):

3 $$\left|AB\right|=\sqrt{{\left({x}_{1}-{x}_{2}\right)}^{2}+{\left({y}_{1}-{y}_{2}\right)}^{2}}$$

It can accurately calculate the displacement of the object between two adjacent frames. Since the time span of one frame is extremely short, according to the definition of instantaneous speed, the average speed during this time can be regarded as the instantaneous speed. It was observed during the research that controlling the frequency of images fed into the speed measurement model has a certain impact on the accuracy of speed estimation. Therefore, based on the frame rate of the video, selecting different time intervals to input images into the model allows for more accurate calculation of the instantaneous speed of the measured object at the corresponding moment according to the actual scenario. The speed calculation formula is:

4 $$v= \frac{\left| AB \right|} {\frac{1}{FPS}}$$

**Fig. 2:**
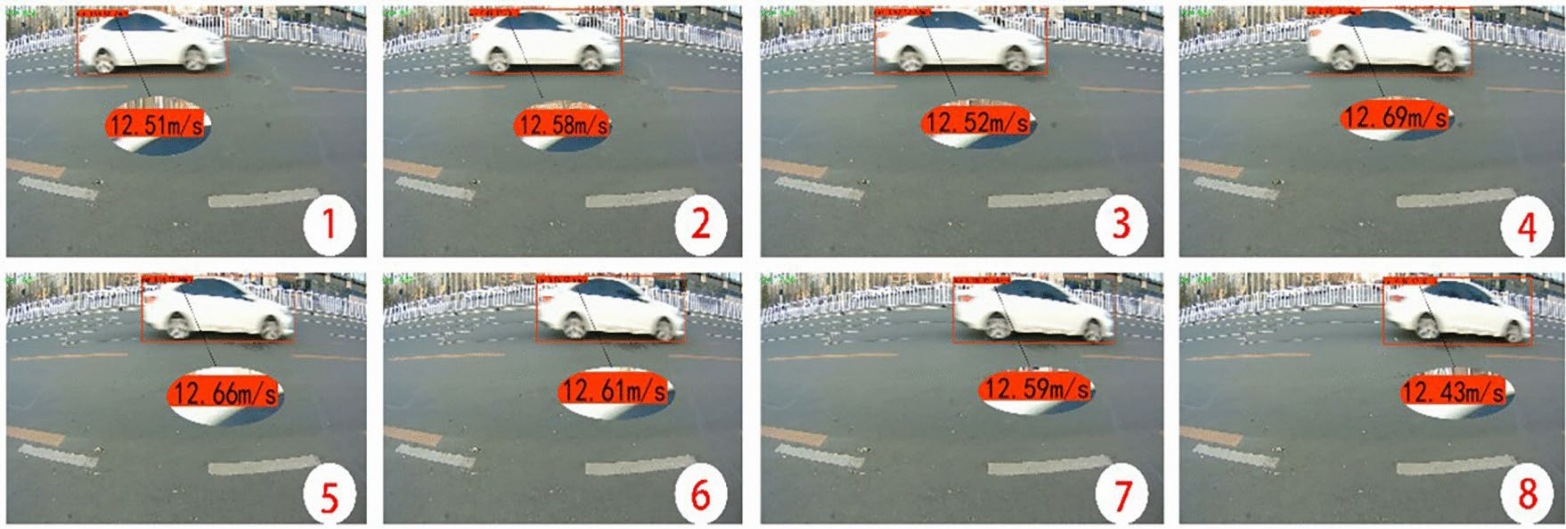
Road scene speed measurement results.

The test results in a real highway environment are shown in Fig. 2. The three parameters above the bounding box are the category of the object, the confidence level, and the instantaneous speed of the object:

### Two-dimensional positioning model

#### Coordinate system transformation in monocular vision

**Fig. 3:**
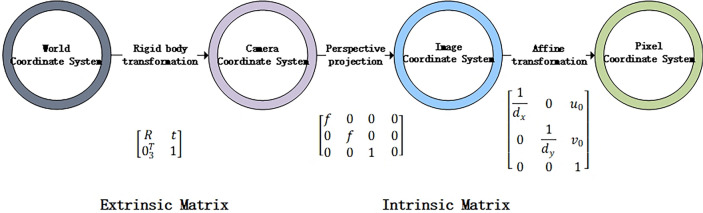
Classic coordinate system transformation diagram.

**Fig. 4:**
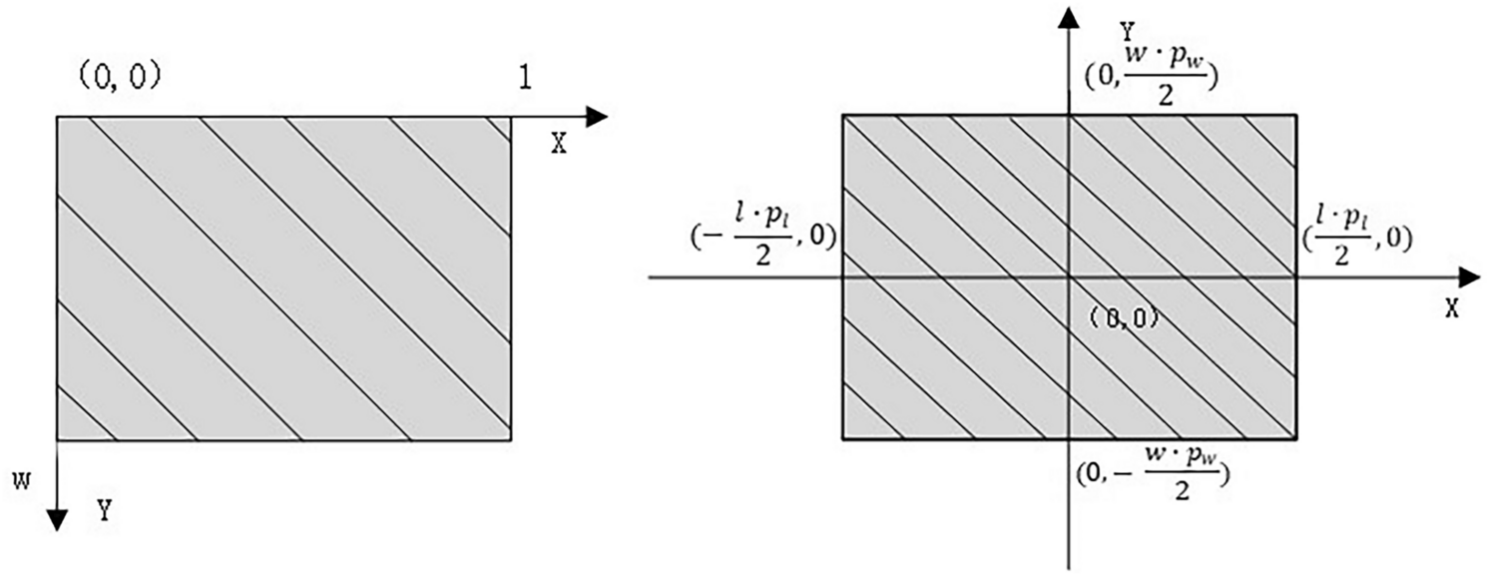
Pixel coordinate system transformation diagram.

In the process of monocular vision imaging, the classic transformation relationships between different coordinate systems are shown in Fig. 3. For the transformation from the pixel coordinate system to the image coordinate system, the origin of the pixel coordinate system on the computer image is at the top-left corner of the image. The *X* and *Y* axes are oriented horizontally to the right and vertically downward, respectively, with the top-left corner as the origin. Therefore, the transformation from the pixel coordinate system to the image coordinate system can be considered as moving the origin of the coordinates to the center of the image. Assuming the image has a length of *l* pixels and a width of *w* pixels, and the length and width of a pixel block are ${p}_{l}$ and ${p}_{w}$, respectively, the transformation formulas for converting from the default pixel coordinates $({x}_{p} ,{y}_{p})$ to physical coordinates $({x}^{\prime},{y}^{\prime})$ are shown in Eqs. (5) and (6). The process of converting from the pixel coordinate system to physical coordinates is illustrated in Fig. 4.

5 $${x}^{\prime}=({x}_{p}-\frac{l}{2}) \cdot {p}_{l}$$

6 $${y}^{\prime}=({y}_{p}-\frac{w}{2}) \cdot {p}_{w}$$

**Fig. 5:**
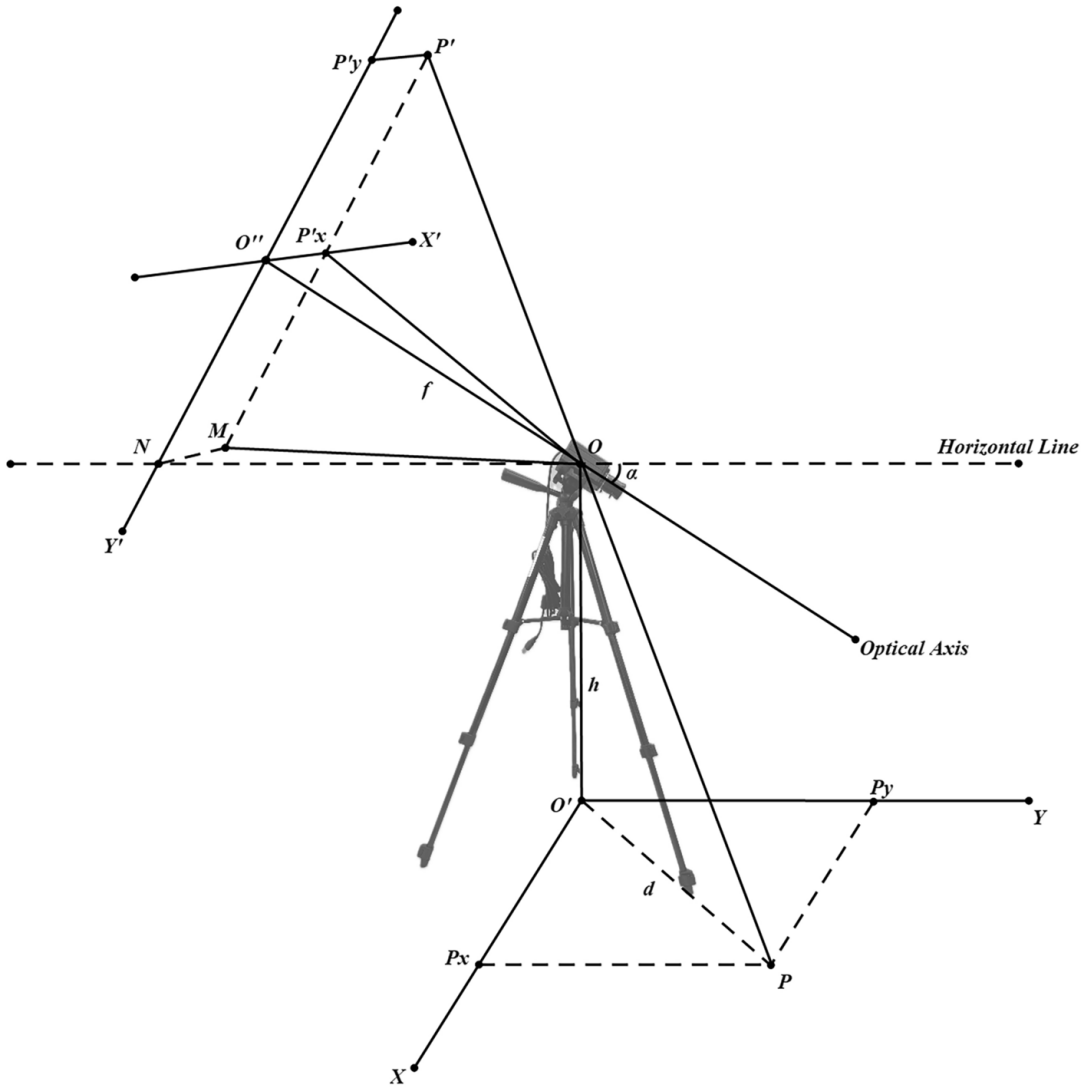
Camera placement and imaging mechanism in the real world. The coordinate system $XO{^{\prime}}Y$ represents the world coordinate system, and the coordinate system ${X}^{{^{\prime}}}{O}^{{^{\prime}}{^{\prime}}}{Y}^{{^{\prime}}}$ is the physical coordinate system. $P(x,y)$ is the point to be measured on the ground, with ${P}_{x}$ as the projection of *P* on the *X*-axis, with a length of *x*, and ${P}_{y}$ as the projection of *P* on the *Y*-axis, with a length of *y*. The segment $O{^{\prime}}P$, with length *d*, represents the straight-line distance from the point *P* to the camera. The optical axis $O{O}^{{^{\prime}}{^{\prime}}}$ passes through the optical center $O$ and is perpendicular to the image coordinate system ${X}^{{^{\prime}}}{O}^{{^{\prime}}{^{\prime}}}{Y}^{{^{\prime}}}$. *α* denotes the angle between the optical axis of the camera and the horizontal line. The distance $OO{^{\prime}}{^{\prime}}$ from the optical center *O* to the origin $O{^{\prime}}{^{\prime}}$ of the image coordinate system is the camera’s focal length *f*, and *h* represents the real-world height of the camera, specifically the distance $OO{^{\prime}}$ from the optical center *O* to the world coordinate system on the ground. After imaging, the point *P* projects to ${P}^{{^{\prime}}}({x}^{{^{\prime}}},{y}^{{^{\prime}}})$ in the physical coordinate system, with ${P}_{x}^{{^{\prime}}}$ as the projection of ${P}^{{^{\prime}}}$ on the ${X}^{{^{\prime}}}$-axis, having a length of ${x}^{{^{\prime}}}$, and ${P}_{y}^{{^{\prime}}}$ as the projection of ${P}^{{^{\prime}}}$ on the ${Y}^{{^{\prime}}}$-axis, with a length of ${y}^{{^{\prime}}}$. Points $M$ and *N* are the intersections of the extended line ${P}^{{^{\prime}}}{P}_{x}^{{^{\prime}}}$ and the extended line ${P}_{y}^{{^{\prime}}}{O}^{{^{\prime}}{^{\prime}}}$ with the horizontal plane where the optical center *O* is located. The ultimate goal of the positioning model is to convert ${P}^{{^{\prime}}}({x}^{{^{\prime}}},{y}^{{^{\prime}}})$ into $P(x,y)$.

For the transformation from the image coordinate system to the camera coordinate system and then to the world coordinate system, this paper establishes a geometric model that simplifies the transformation between the three coordinate systems into the imaging model shown in Fig. 5. The placement posture and imaging mechanism of the camera in this model are shown in Fig. 5:

#### Two-dimensional positioning

According to the pinhole imaging principle, this paper assumes that the distance from the optical center $O$ to the origin $O{^{\prime}}{^{\prime}}$ of the image plane is the focal length *f* of the camera. Based on the camera imaging model and spatial geometric relationships proposed above, the formulas for calculating the world coordinates *x* and *y* of the feature points can be derived as Eqs. (7) and (8):

7 $$x=\frac{h\cdot \left({x}^{{^{\prime}}2}+{f}^{2}-{y}{\prime}\cdot f\cdot \mathit{tan}\alpha \right)\cdot sin\beta }{\sqrt{{x}^{{^{\prime}}2}+{f}^{2}}\cdot \left(f\cdot \mathit{tan}\alpha +{y}{\prime}\right)}$$

8 $$y=\frac{h\cdot \left({x}^{{^{\prime}}2}+{f}^{2}-{y}{\prime}\cdot f\cdot \mathit{tan}\alpha \right)\cdot cos\beta }{\sqrt{{x}^{{^{\prime}}2}+{f}^{2}}\cdot \left(f\cdot \mathit{tan}\alpha +{y}{\prime}\right)}$$

The derivation process is as follows:

Since the horizontal plane is parallel to the ground plane:

9 $${\angle }{OPO}^{{^{\prime}}}={\angle }{P}^{{^{\prime}}}OM={\angle }{P}^{{^{\prime}}}O{P}_{x}^{{^{\prime}}}+{\angle }{P}_{x}^{{^{\prime}}}OM$$

In Δ $N{O}^{{^{\prime}}{^{\prime}}}O,\text{ since }N{O}^{{^{\prime}}{^{\prime}}}\perp O{O}^{{^{\prime}}{^{\prime}}},$ we have:

10 $$\begin{array}{c}{O}^{{^{\prime}}{^{\prime}}}N=f\cdot \mathit{tan}\alpha\end{array}$$

In $\Delta {O}^{{^{\prime}}{^{\prime}}}{P}_{x}^{{^{\prime}}}O, {O}^{{^{\prime}}{^{\prime}}}{P}_{x}^{{^{\prime}}}={x}{\prime},OO{^{\prime}}{^{\prime}}\perp O{^{\prime}}{^{\prime}}{P}_{x}^{{^{\prime}}}$, we have:

11 $$\begin{array}{c}O{P}_{x}^{{^{\prime}}}=\sqrt{{x}^{{^{\prime}}2}+{f}^{2}}\end{array}$$

Since ${P}_{x}^{{^{\prime}}}M={O}^{{^{\prime}}{^{\prime}}}N\text{ and in }\Delta {P}_{x}^{{^{\prime}}}OM, {OP}_{x}^{{^{\prime}}}\perp {P}_{x}^{{^{\prime}}}M$, we have:

12 $$\begin{array}{c}\angle {P}_{x}^{{^{\prime}}}OM={\mathit{tan}}^{-1}\frac{{P}_{x}^{{^{\prime}}}M}{O{P}_{x}^{{^{\prime}}}}={\mathit{tan}}^{-1}\frac{f\cdot \mathit{tan}\alpha }{\sqrt{{x}^{{^{\prime}}2}+{f}^{2}}}\end{array}$$

In $\Delta {P}^{{^{\prime}}}O{P}_{x}^{{^{\prime}}}, O{P}_{x}^{{^{\prime}}}\perp P{^{\prime}}{P}_{x}^{{^{\prime}}}$, we have:

13 $$\begin{array}{c}\angle {P}^{{^{\prime}}}O{P}_{x}^{{^{\prime}}}={\mathit{tan}}^{-1}\frac{{P}^{{^{\prime}}}{P}_{x}^{{^{\prime}}}}{O{P}_{x}^{{^{\prime}}}}={\mathit{tan}}^{-1}\frac{{y}{\prime}}{\sqrt{{x}^{{^{\prime}}2}+{f}^{2}}}\end{array}$$

In $\Delta OP{O}^{{^{\prime}}}, PO{^{\prime}}\perp OO{^{\prime}}$, we have:

14 $$\begin{array}{c}\angle {OPO}^{{^{\prime}}}= {\mathit{tan}}^{-1}\frac{O{O}^{{^{\prime}}}}{{O}^{{^{\prime}}}P}={\mathit{tan}}^{-1}\frac{h}{d}\end{array}$$

Combining Eqs. (8), (11), (12), (13), we get:

15 $$\begin{array}{c}{\mathit{tan}}^{-1}\frac{f\cdot \mathit{tan}\alpha }{\sqrt{{x}^{{^{\prime}}2}+{f}^{2}}}+{\mathit{tan}}^{-1}\frac{{y}{\prime}}{\sqrt{{x}^{{^{\prime}}2}+{f}^{2}}}={\mathit{tan}}^{-1}\frac{h}{d}\end{array}$$

Using the tangent addition formula, we have:

16 $$\begin{array}{c}{\mathit{tan}}^{-1}\frac{f\cdot \mathit{tan}\alpha }{\sqrt{{x}^{{^{\prime}}2}+{f}^{2}}}+{\mathit{tan}}^{-1}\frac{{y}{\prime}}{\sqrt{{x}^{{^{\prime}}2}+{f}^{2}}}={\mathit{tan}}^{-1}\frac{\frac{f\cdot \mathit{tan}\alpha }{\sqrt{{x}^{{^{\prime}}2}+{f}^{2}}}+\frac{{y}{\prime}}{\sqrt{{x}^{{^{\prime}}2}+{f}^{2}}}}{1-\frac{f\cdot \mathit{tan}\alpha }{\sqrt{{x}^{{^{\prime}}2}+{f}^{2}}}\cdot \frac{{y}{\prime}}{\sqrt{{x}^{{^{\prime}}2}+{f}^{2}}}}\\ ={\mathit{tan}}^{-1}\frac{\sqrt{{x}^{{^{\prime}}2}+{f}^{2}}\cdot \left(f\cdot \mathit{tan}\alpha +{y}{\prime}\right)}{{x}^{{^{\prime}}2}+{f}^{2}-{y}{\prime}\cdot f\cdot \mathit{tan}\alpha }={\mathit{tan}}^{-1}\frac{h}{d}\end{array}$$

Thus:

17 $$\frac{\sqrt{{x}^{{^{\prime}}2}+{f}^{2}}\cdot \left(f\cdot \mathit{tan}\alpha +{y}{\prime}\right)}{{x}^{{^{\prime}}2}+{f}^{2}-{y}{\prime}\cdot f\cdot \mathit{tan}\alpha }=\frac{h}{d}$$

From Eq. (17), we can derive:

18 $$d=\frac{h\cdot \left({x}^{{^{\prime}}2}+{f}^{2}-{y}{\prime}\cdot f\cdot \mathit{tan}\alpha \right)}{\sqrt{{x}^{{^{\prime}}2}+{f}^{2}}\cdot \left(f\cdot \mathit{tan}\alpha +{y}{\prime}\right)}$$

Equation (18) is the ranging formula.

In constructing the world coordinate system, the *Y*-axis is parallel to the main optical axis of the camera, and the *X*-axis is parallel to *MN*. Since *MO* and ${O}{\prime}P$ are the projections of ${P}{\prime}P$ on the horizontal plane of the camera and the ground respectively, *MO* is parallel to ${O}{\prime}P$. Therefore, we get:

19 $$\Delta OMN\sim \Delta {O}^{{^{\prime}}}P{P}_{y}$$

From the geometric model, we know:

20 $${OO}^{{^{\prime}}{^{\prime}}}=f$$

21 $$MN={O}^{{^{\prime}}}{^{\prime}}{P}_{x}^{{^{\prime}}}={x}{\prime}$$

22 $$ON=\frac{f}{cos\alpha }$$

23 $$OM=\sqrt{\frac{{f}^{2}}{{cos\alpha }^{2}}+{x}^{{^{\prime}}2}}$$

Combining (20), (21), (22) and (23), we get (24), (25):

24 $$cos\beta =\frac{f}{\sqrt{{f}^{2}+{x}^{{^{\prime}}2}\cdot {cos\alpha }^{2}}}$$

25 $$\begin{array}{c}sin\beta =\frac{{x}{\prime}\cdot cos\alpha }{\sqrt{{f}^{2}+{x}^{{^{\prime}}2}\cdot {cos\alpha }^{2}}}\end{array}$$

Combining Eqs. (18), (24), and (25) results in the calculation formulas for *x* and *y*, as Eqs. (7) and (8). Some experimental results are shown in Table 3:

**Table 3:** Positioning results and accuracy.

Feature	X (mm)	Y (mm)	Compensation X	Compensation Y	X accuracy	Y accuracy
1	− 540.000	360.000	− 556.806	373.571	0.968	0.962
2	360.000	360.000	283.492	282.392	0.787	0.784
3	− 300.000	420.000	− 218.236	305.243	0.727	0.727
4	− 300.000	480.000	− 238.168	379.969	0.793	0.791
5	− 120.000	540.000	− 83.7065	373.148	0.697	0.691
6	720.000	540.000	848.482	637.758	0.822	0.819
7	780.000	600.000	950.275	732.467	0.781	0.779
8	600.000	660.000	700.929	767.472	0.832	0.837
9	600.000	720.000	718.530	858.457	0.803	0.807

According to the experimental results in Table 3, there is a significant error between the calculated feature point coordinates and the actual situation. Therefore, the next section will explore the causes of these errors and improve positioning accuracy by optimizing the camera calibration method, recalibrating the focal length, and establishing a coordinate error compensation mechanism.

#### Camera calibration

**Fig. 6:**
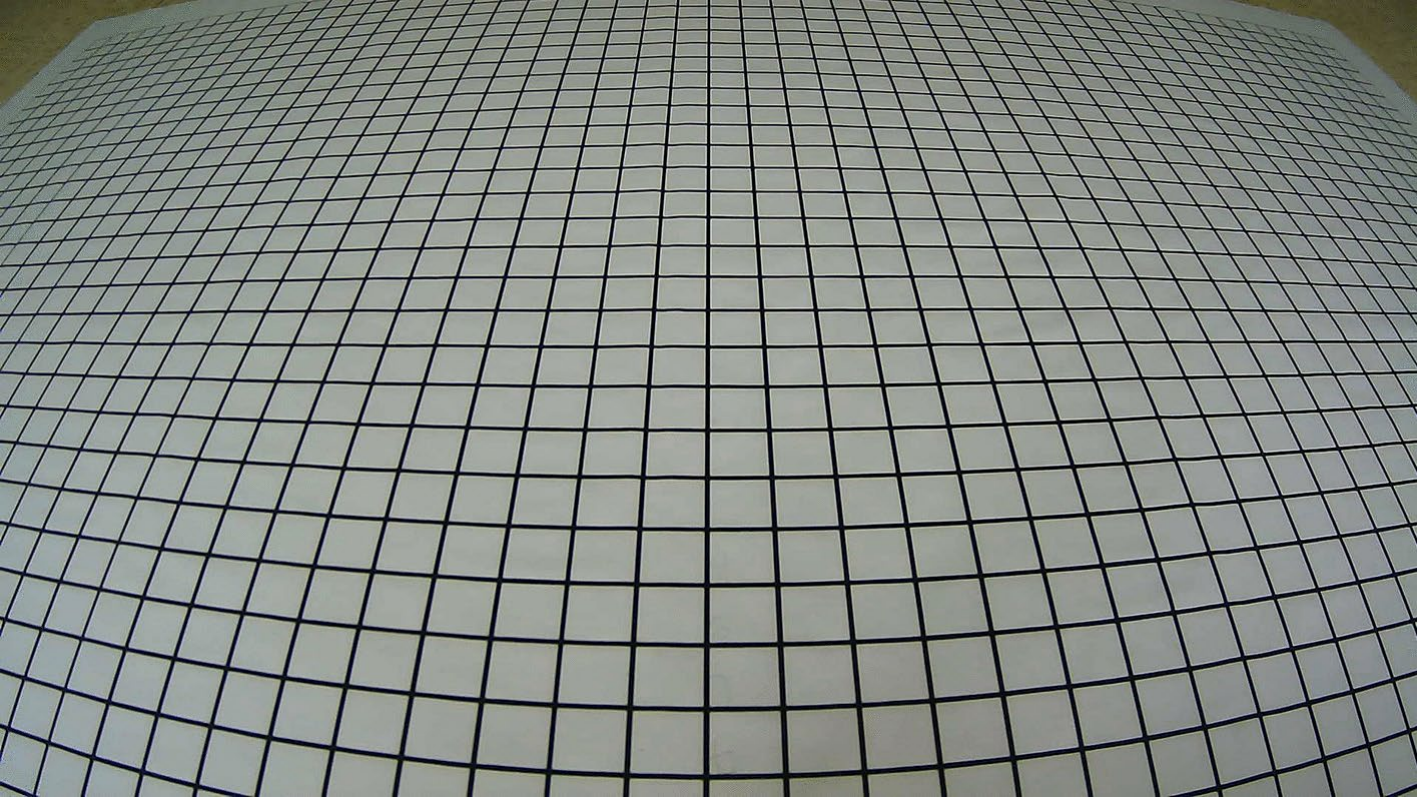
Camera calibration environment.

**Fig. 7:**
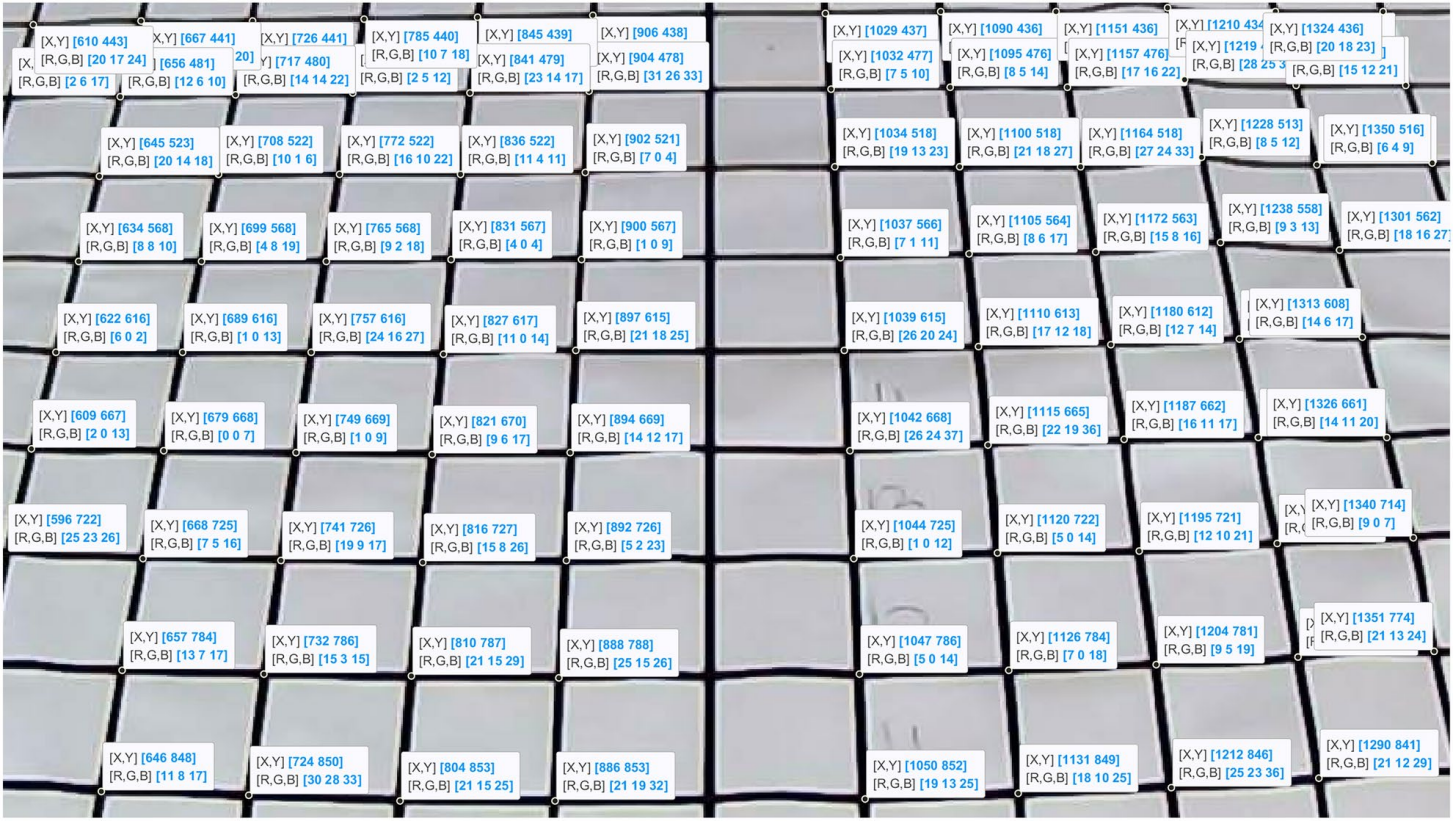
Example of partial feature point extraction.

In the formulas for calculating the world coordinates *x* and *y* of the feature points mentioned above, the focal length *f* is the most important internal parameter of the camera and the only internal parameter required in this study. It needs to be determined through camera calibration. Camera calibration is crucial to ensure the accuracy of data calculations for the object being measured in the real world. To determine the camera’s internal and external parameters and map the captured images to the world coordinate system, the camera should be calibrated before data collection. First, place the grid paper A on the ground, as shown in Fig. 6:

When calibrating the camera, with the height and tilt angle of the camera known, the focal length *f* can be calculated after extracting the pixel coordinates of the target points on the grid paper using MATLAB’s Datatip function. Partial results of the extracted points are shown in Fig. 7.

In the method proposed by Xue Lixia et al.^23^, the equation for calculating f yields four different values of *f*, including one normal value and three abnormal values containing imaginary parts. To obtain a reliable focal length value, the abnormal values need to be excluded. However, this solving process is relatively complex, and the exclusion of abnormal values also increases the workload. Therefore, by derivation, another formula for solving *f* can be obtained by combining Eqs. (7), (8), (24), and (25):

26 $$\begin{array}{c}f=\frac{{x}{\prime}\cdot y\cdot cos\alpha }{x}\end{array}$$

The focal length *f* values calculated from different measured points vary, which means that the focal length *f* is related to the pixel coordinates $(x,y)$. Therefore, by treating the focal length *f* as the dependent variable and the physical coordinates ${x}{\prime}$ and ${y}{\prime}$ as the independent variables, a polynomial fitting method can be used to obtain a fitting polynomial in the form of Eq. (27). The fitting results have the following statistics: R-square = 0.9992, SSE = 0.07959, RMSE = 0.009138. After obtaining the fitting equation, the physical coordinates of the measured points can be substituted in to calculate the corresponding focal length *f* for that point.

27 $$\begin{array}{c}f\left(x,y\right)=p00+p10*x+p01*y+p20*{x}^{2}+p11*x*y+\\ p02*{y}^{2}+p30*{x}^{3}+p21*{x}^{2}*y +p12*x*{y}^{2}\end{array}$$

**Fig. 8:**
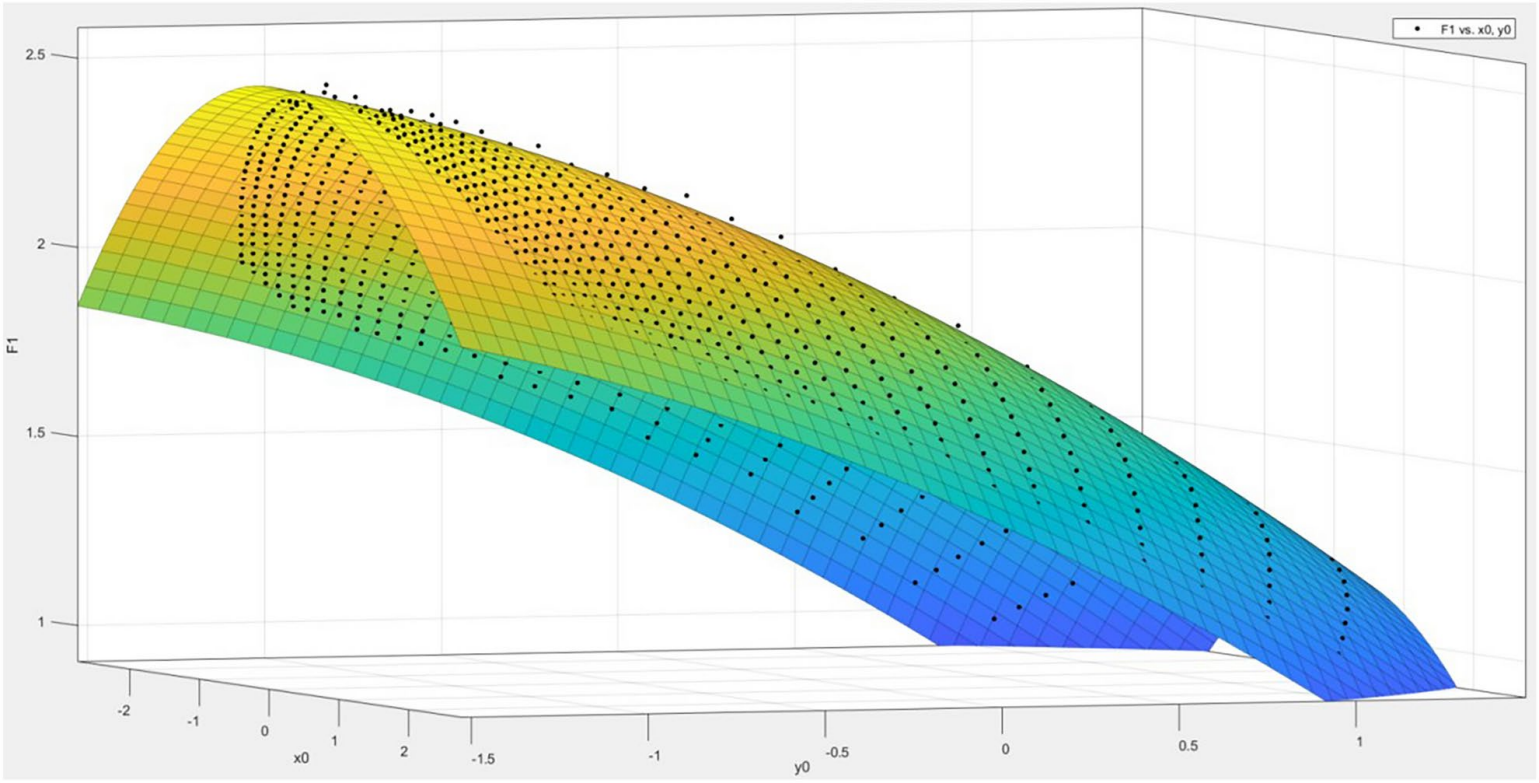
Focal length fitting effect.

The resulting fitting effect diagram is shown in Fig. 8:

#### Establishing an error compensation mechanism

**Fig. 9:**
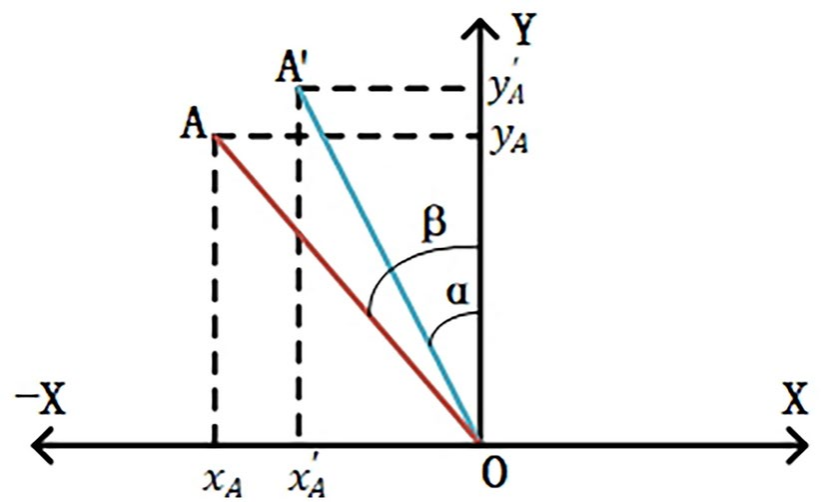
Error vector diagram.

Based on the previously mentioned calculation results, it is found that the calculated world coordinates $(x, y)$ have certain discrepancies from the actual world coordinates. Assume that the measured point is *A*, with actual world coordinates $A({x}_{A}, {y}_{A})$. The world coordinates calculated using Eqs. (7) and (8) are $A{^{\prime}}({x}_{A}^{{^{\prime}}}, {y}_{A}^{{^{\prime}}})$. Further analysis reveals that the distances from both *A* and $A{^{\prime}}$ to the origin of the world coordinate system are equal, $AO={A}^{{^{\prime}}}O$, indicating that the two points form a concentric circle centered at *O* with an angular deviation of ${\angle }b-a$. Therefore, $[({x}_{A}-{x}_{A}^{{^{\prime}}}), \left({y}_{A}-{y}_{A}^{{^{\prime}}}\right)]$ represents the coordinate error between the calculated and actual values. This situation is illustrated in Fig. 9.

To address the discrepancies between calculated values and actual values, polynomial regression is used for fitting. Let $[({x}_{A}-{x}_{A}^{{^{\prime}}}), \left({y}_{A}-{y}_{A}^{{^{\prime}}}\right)]$ represent the error between calculated and actual values. Polynomial fitting is used to obtain the error distribution corresponding to different pixel coordinates, where p represents the regression coefficients. Ultimately, the error values are compensated into the calculated values to obtain compensated coordinates $A{^{\prime}}{^{\prime}}({x}_{A}^{{^{\prime}}{^{\prime}}}, {y}_{A}^{{^{\prime}}{^{\prime}}})$. Through tens of thousands of experiments under various conditions and for different test points, this error compensation method has shown excellent results.

**Fig. 10:**
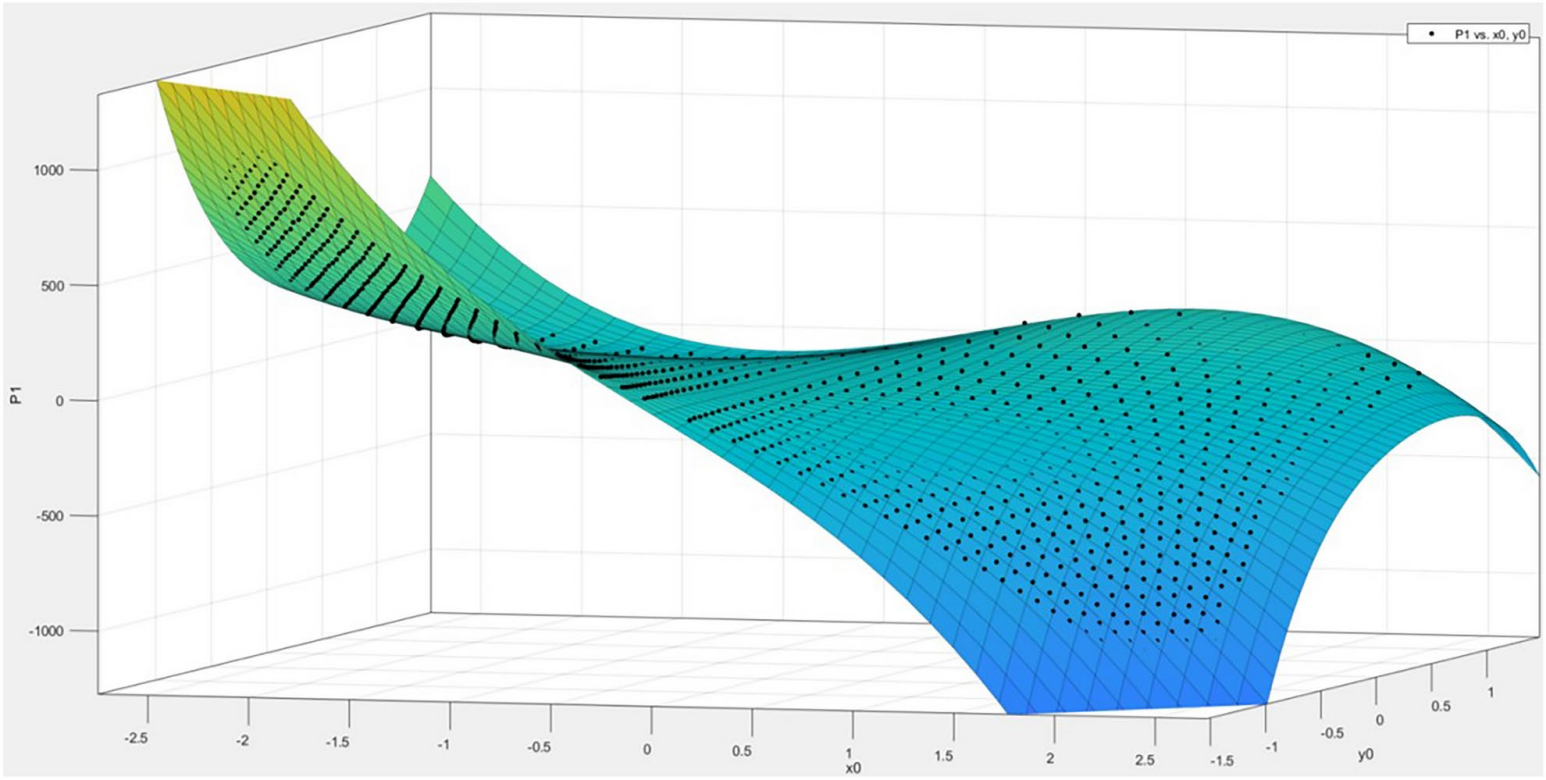
Error between horizontal coordinates and pixel coordinates $T(x,y)$ fitting effect diagram.

In the error compensation method, error values ${t}_{1}={x}_{A}-{x}_{A}^{{^{\prime}}}$ and ${t}_{2}={y}_{A}-{y}_{A}^{{^{\prime}}}$ are fitted with the physical coordinates *x*, *y* of the target point under ${x}^{3}, {y}^{5}$ conditions. The fitting effect of $T(x,y)$ for the horizontal coordinate is shown in Fig. 10:

The obtained fitted formula is (28), with the statistical metrics being R-square = 0.9994, SSE = 1.15e + 05, and RMSE = 11.04.

28 $$\begin{array}{c}T\left(x,y\right)= p00 + p10*x + p01*y + p20*{x}^{2}+ p11*x*y + p02*{y}^{2}+ \\ p30*{x}^{3}+ p21*{x}^{2}*y + p12*x*{y}^{2}+ p03*{y}^{3}+ \\ p31*{x}^{3}*y + p22*{x}^{2}*{y}^{2}+ p13*x*{y}^{3}+ p04*{y}^{4}+ \\ p32*{x}^{3}*{y}^{2}+ p23*{x}^{2}*{y}^{3}+ p14*x*{y}^{4}+ p05*{y}^{5}\end{array}$$

**Fig. 11:**
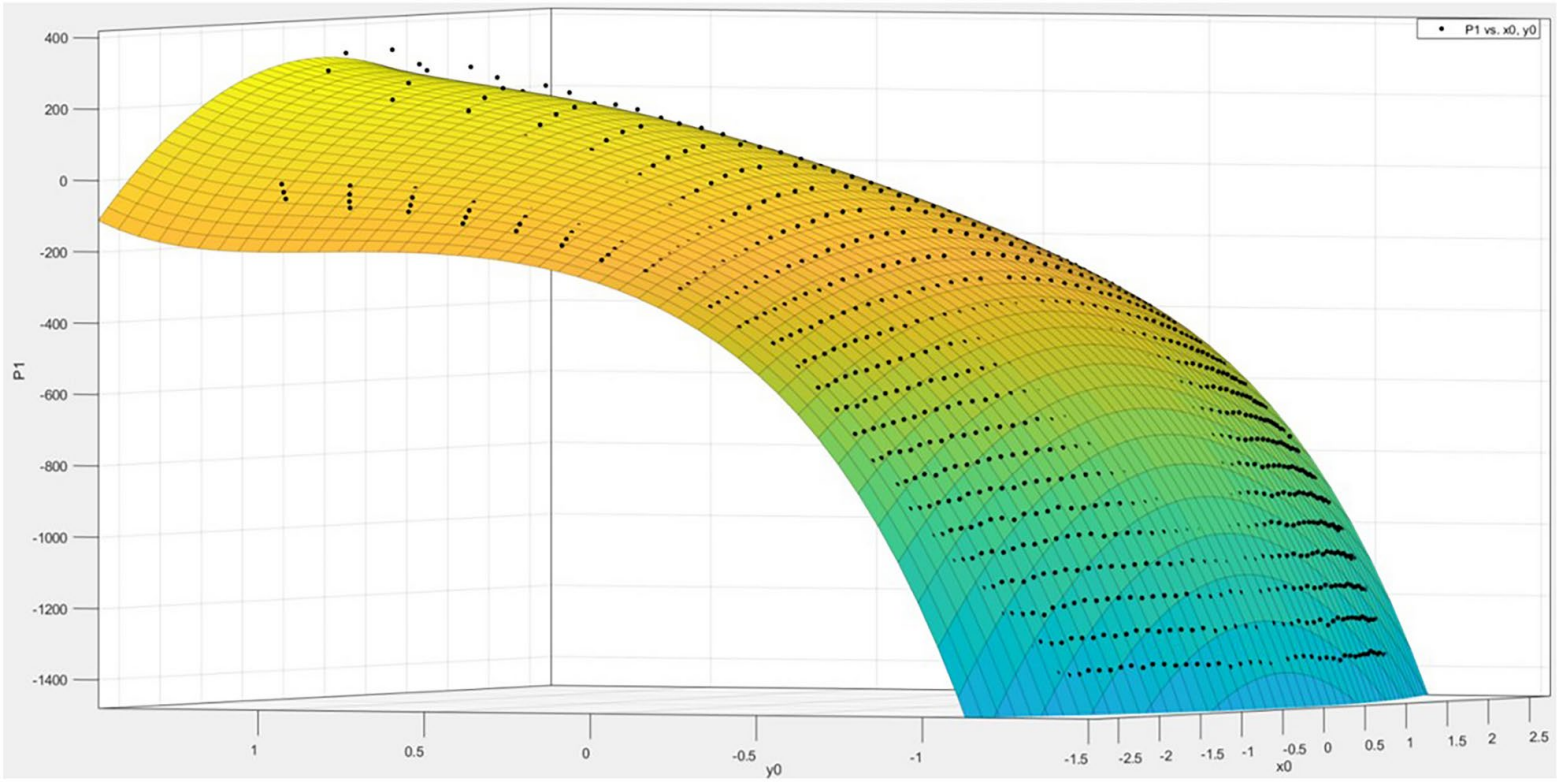
Fitting result of $T(x,y)$ for the vertical coordinates error and image coordinates.

The fitting result of $T(x,y)$ for the vertical coordinates under the conditions of ${x}^{3}, {y}^{5}$ is shown in Fig. 11.

The obtained fitting formula is (29), with the statistical measures as follows: R-square = 0.9989, SSE = 2.083e + 05, and RMSE = 14.85.

29 $$\begin{array}{c}T\left(x,y\right)= p00 + p10*x + p01*y + p20*{x}^{2}+ p11*x*y + p02*{y}^{2}+ \\ p30*{x}^{3}+ p21*{x}^{2}*y + p12*x*{y}^{2}+ p03*{y}^{3}+ \\ p31*{x}^{3}*y + p22*{x}^{2}*{y}^{2}+ p13*x*{y}^{3}+ p04*{y}^{4}+ \\ p32*{x}^{3}*{y}^{2}+ p23*{x}^{2}*{y}^{3}+ p14*x*{y}^{4}+ p05*{y}^{5}\end{array}$$

## Results and discussion

This section describes in detail the equipment, experimental methods, and results analysis used to measure vehicle speed from video data. Each set of experiments was conducted in real road scenarios. The camera used had a resolution of 1920 × 1080 and a frame rate of 30 Hz. During the experiments, a laser rangefinder was used to measure the height and tilt angle of the camera from the ground. In the pre-camera calibration process, a checkerboard A with squares of 6 cm x 6 cm was used. Once the calibration was completed, changing the experimental scene did not affect the measurement results. The data processing was carried out on a laptop with the following configuration: CPU: Intel Core i7-8750H 2.20 GHz, Memory RAM: 16 GB DDR4 2666 MHz (8 GB × 2), GPU: NVIDIA GeForce GTX 1050Ti.

### Single target experimental results analysis

#### Impact of different heights and tilt angles on speed measurement accuracy

To verify the accuracy and effectiveness of the speed measurement method proposed in this study, tens of thousands of experiments were conducted under different camera heights and tilt angles, with the target number controlled to a single object. The camera installation position was considered the origin $(\text{0,0})$, and the height and tilt angle of the camera remained unchanged within the same set of experiments. The verification process selected effective frames in the video containing moving objects. The real-world displacement of the vehicle between two frames was manually measured to calculate its actual speed, which was then compared with the automatically measured speed. Table 4 presents the minimum accuracy, average accuracy, and frame rate (FPS) of speed measurements at different heights and angles.

**Table 4:** Experimental results at different heights and angles.

Experiment	Height	Tilt angle	Minimum accuracy	Average accuracy	FPS	Average accuracy
1	706 mm	37°	95.9%	97.6%	9.6	1298
2	706 mm	42°	95.1%	97.0%	9.7	1509
3	706 mm	46°	95.9%	97.3%	9.6	1038
4	750 mm	37°	95.9%	97.9%	9.8	1325
5	750 mm	42°	96.1%	98.0%	9.5	1407
6	750 mm	46°	95.8%	97.1%	9.6	1113
7	780mm	37°	95.6%	97.5%	9.7	1169
8	780mm	42°	96.2%	98.2%	9.7	1039
9	780mm	46°	95.8%	97.6%	9.8	1488

The accuracy calculation method is as follows: the automatically calculated speed by the program is considered the predicted speed ${v}_{1}$, and the manually measured actual displacement of the object is used to obtain the true speed ${v}_{2}$. The predicted and true speeds are then substituted into Eqs. (30) and (31) to calculate the average accuracy and minimum accuracy:

30 $$Accuracy=\frac{\sum_{i=1}^{n}1-ABS\left(\frac{{v}_{1}-{v}_{2}}{{v}_{1}}\right)}{n}$$

31 $$in Accuracy=min\left(1-ABS\left(\frac{{v}_{1}-{v}_{2}}{{v}_{1}}\right)\right)$$

Under the same tilt angle, the experimental results showed slight fluctuations with increasing camera height. For example, under a 46° tilt angle, the experiments with a height of 780 mm (experiments 7, 8, 9) had a slightly higher average accuracy than the other two heights. The average accuracy varied slightly under different tilt angle conditions. Under a 42° tilt angle (experiments 2, 5, 8), the average accuracy was relatively high, while the differences were smaller at 37° and 46°. The frame rate remained relatively consistent across different heights and tilt angles, ranging from 9.5 to 9.8, indicating stable system processing speed under various experimental conditions. Based on the analysis of results across different heights and angles, it can be concluded that the camera height and tilt angle have a minimal impact on system performance. Therefore, in practical applications, the appropriate camera height and tilt angle can be chosen according to the specific requirements of the scene, without concerns about adverse effects on accuracy due to external parameters.

#### Impact of different lighting conditions on speed measurement accuracy

**Fig. 12:**
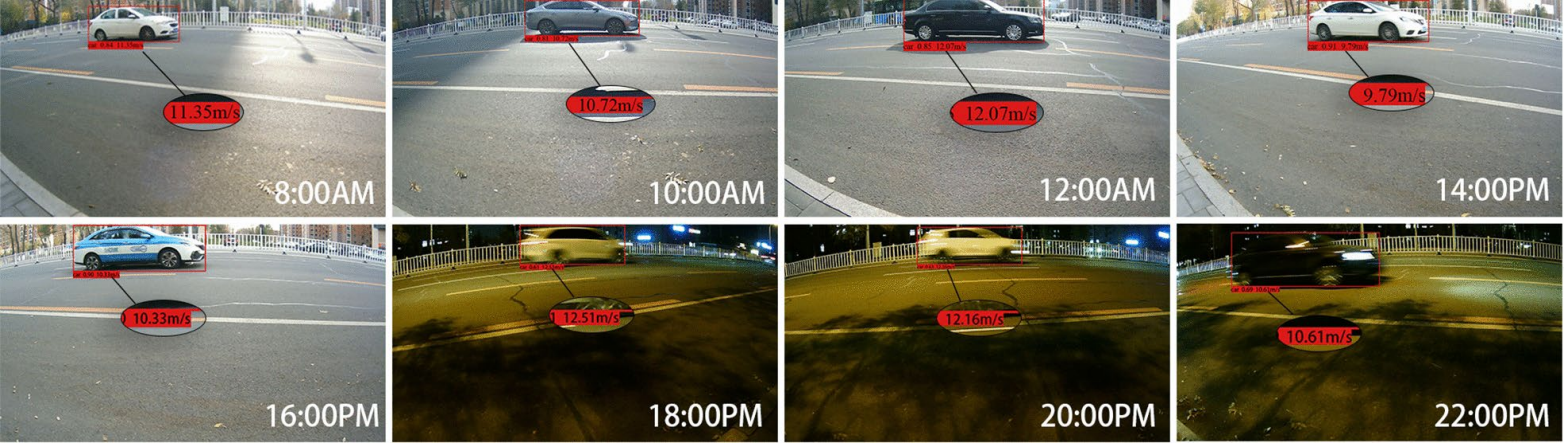
Detection results under different lighting conditions.

This study analyzed the impact of different lighting conditions on measurement results. Experiments were conducted every two hours from 8:00 AM to 10:00 PM to observe the performance of this method in speed measurement under varying lighting intensities. Figure 12 shows the detection results under different lighting conditions.

Table 5 summarizes the speed measurement accuracy and FPS under different time periods:

**Table 5:** Speed measurement results at different time intervals.

Time	Minimum accuracy	Average accuracy	FPS
8:00AM	96.7%	97.9%	9.8
10:00AM	96.4%	98.1%	9.8
12:00AM	96.1%	97.6%	9.8
14:00PM	96.5%	97.9%	9.8
16:00PM	96.4%	97.7%	9.8
18:00PM	95.9%	97.6%	9.8
20:00PM	96.4%	97.4%	9.8
22:00PM	95.8%	97.1%	9.8

The results indicate that this method maintains high measurement accuracy and a stable FPS across different lighting conditions, though changes in lighting intensity do have some impact on speed measurement precision. During daytime hours with stronger lighting (such as from 10:00 AM to 4:00 PM), the system’s average accuracy remains above 97.6%, with minimum accuracy above 96.1%, indicating stable measurements under adequate lighting. Under lower lighting conditions (e.g., from 6:00 PM to 10:00 PM), accuracy fluctuates slightly. As lighting diminishes, slight detection omissions occur, causing minor decreases in speed measurement accuracy for some frames. This reduction is attributed to increased image noise under low light, which slightly lowers target detection accuracy, though it remains generally high. Additionally, FPS remains consistent across time periods, showing that changes in lighting have minimal impact on processing speed but may affect detection accuracy. Thus, in practical applications, supplementary lighting could be considered under low-light or nighttime conditions to enhance system robustness and measurement accuracy.

Overall, this method demonstrates strong speed measurement capabilities under various lighting conditions throughout the day, making it highly adaptable to complex lighting environments and promising for real-world applications.

#### Impact of different time intervals on speed measurement accuracy

This study further analyzes the impact of different time intervals (1-frame interval, 3-frame interval, and 5-frame interval) on speed measurement accuracy. Table 6 presents the missed detection rate, minimum accuracy, and average accuracy at different time intervals:

**Table 6:** Experimental results for speed calculation at different time intervals.

Time interval (frames)	Miss rate	Minimum accuracy	Average accuracy	FPS
1	3.1074%	95.9%	97.6%	9.8
3	2.7113%	95.4%	98.0%	9.8
5	2.4082%	97.4%	98.5%	9.8

Analysis of the experimental results reveals that as the time interval increases, the missed detection rate gradually decreases, while both minimum accuracy and average accuracy improve. At a 1-frame interval, the minimum accuracy is 95.1%, which increases to 97.4% at a 5-frame interval. This improvement is due to the distribution of errors across multiple frames as the interval lengthens, thus reducing the impact of missed detections on speed measurement accuracy. Additionally, longer intervals can improve video processing efficiency; for example, detecting every 3 frames allows processing three seconds’ worth of video in one second. However, increasing the interval may lead to some key frames being skipped, potentially affecting the precision of speed estimation. Therefore, the choice of time interval should balance the accuracy and real-time requirements of the specific application.

**Fig. 13:**
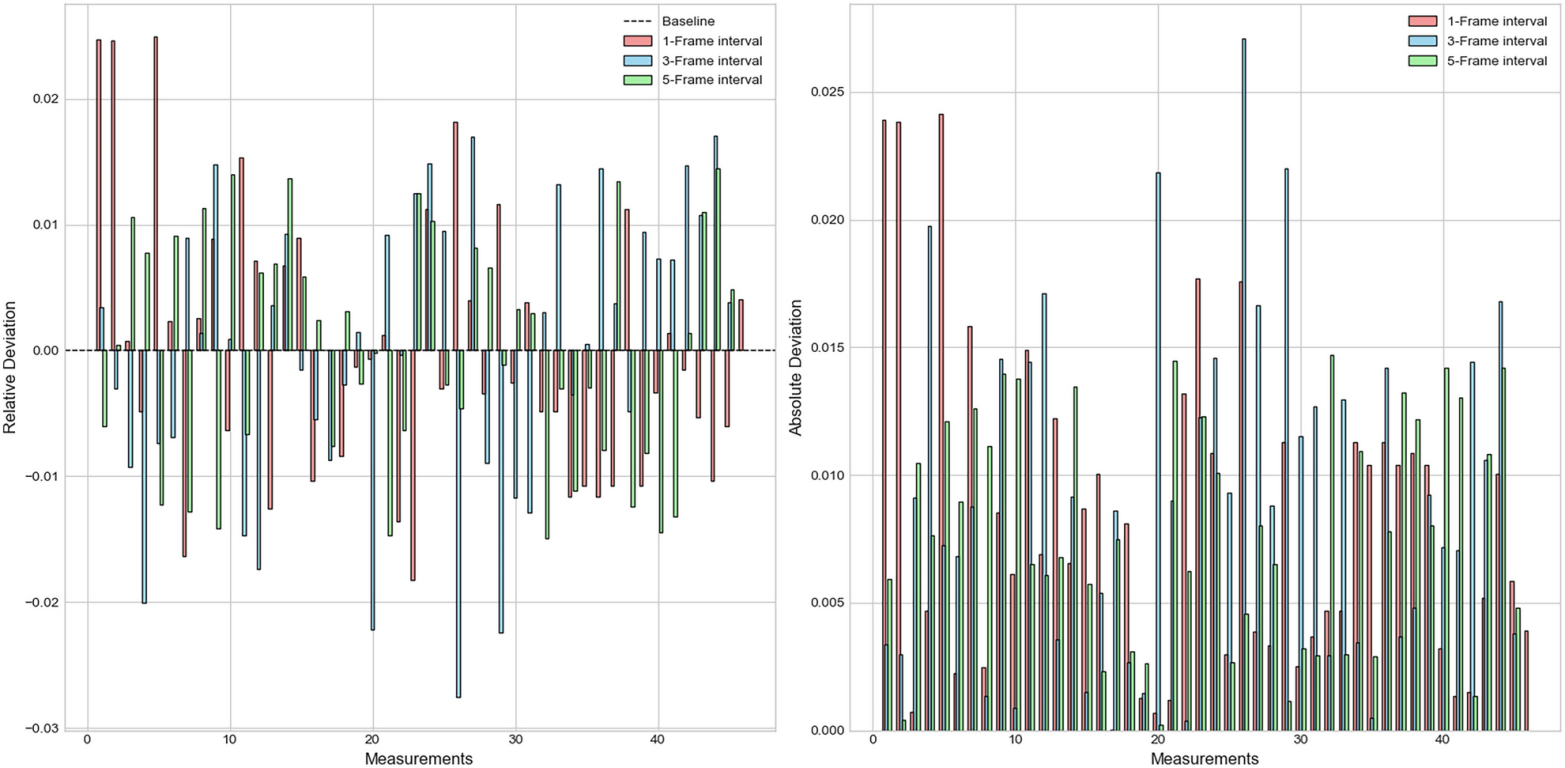
Calculation results of relative and absolute errors at different time intervals.

Figure 13 shows the calculation results of relative and absolute deviations under different time intervals, the left image shows the relative deviation, and the right image shows the absolute deviation:

Analyzing the absolute and relative deviations of the experimental data reveals that, in terms of relative deviation, the 3-frame interval and 5-frame interval show overall smaller deviations, indicating better stability and consistency in the measurements. In contrast, the 1-frame interval shows relatively larger relative deviations, which may suggest greater fluctuation in certain measurements and some irregularity, leading to reduced stability. Regarding absolute deviation, both the 3-frame interval and 5-frame interval exhibit generally smaller absolute deviations, with most measurements concentrated around the mean, indicating higher measurement accuracy. Although there are some measurements with larger deviations in the 3-frame interval, their overall impact is minimal. Considering both absolute and relative deviations, the 5-frame interval performs best in terms of accuracy and consistency, followed by the 1-frame interval and 3-frame interval, which also demonstrate relatively good measurement accuracy and consistency.

**Fig. 14:**
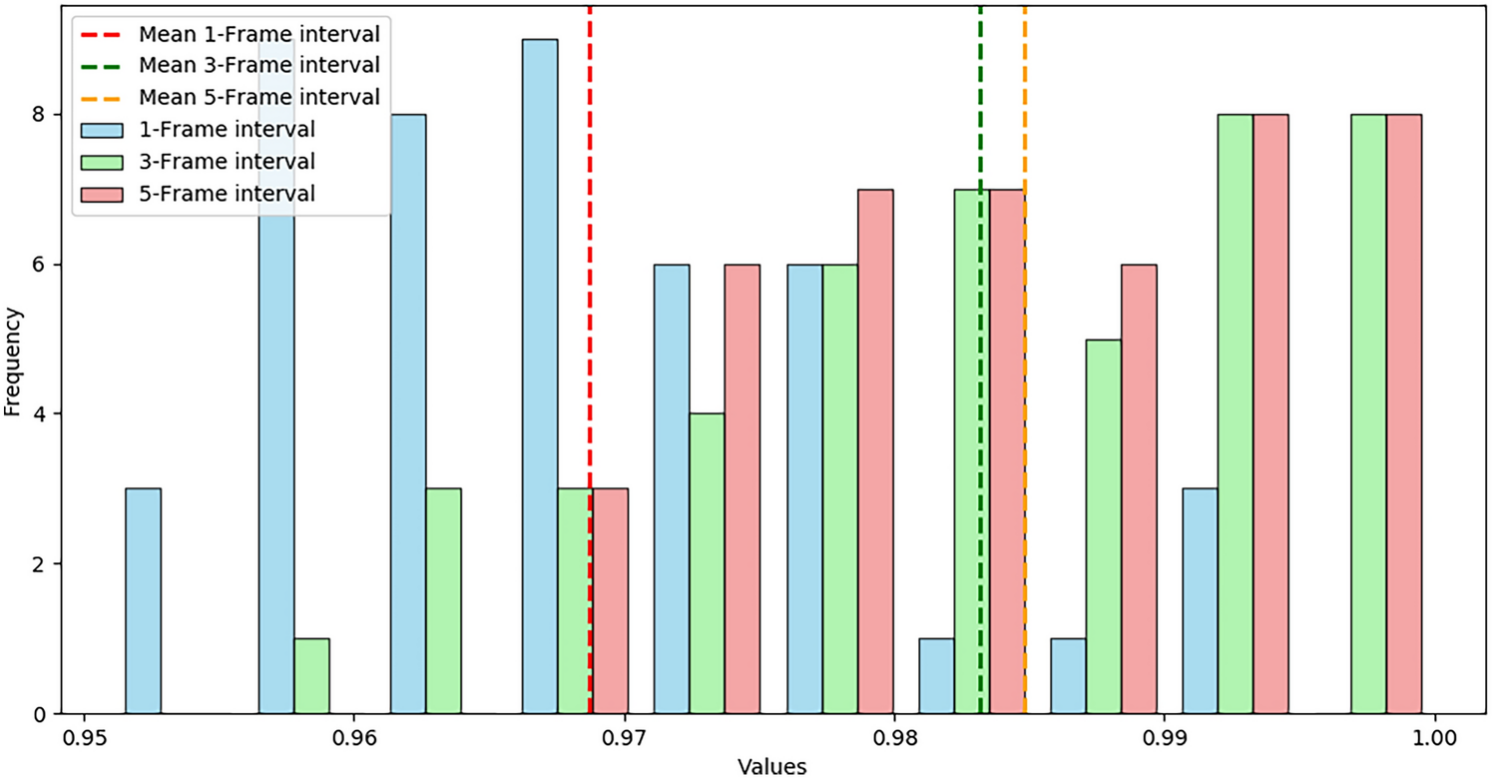
Stacked histogram at different time intervals.

**Fig. 15:**
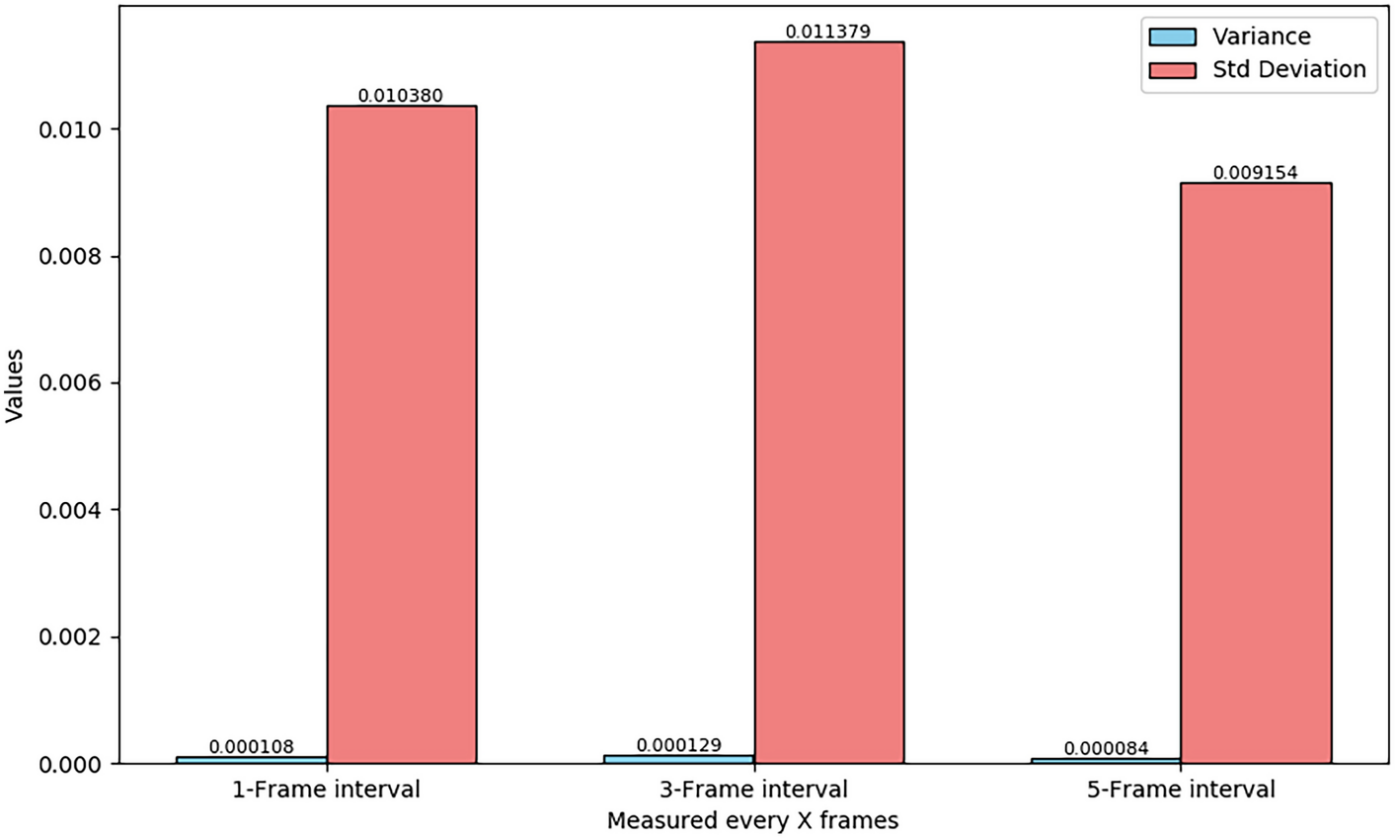
Variance and standard deviation at different time intervals.

Figures 14 and 15 show the variance and standard deviation analysis results for different time intervals:

By observing the stacked histogram, it can be seen that measurements under the 5-frame interval are more concentrated, indicating that longer intervals provide more stable measurements. In contrast, the 1-frame interval and 3-frame interval show a wider distribution range, suggesting greater measurement fluctuation at shorter time intervals. This distribution difference suggests that longer measurement intervals can yield more stable results, while shorter intervals may increase variability in measurement results. The variance and standard deviation bar chart in Fig. 15 further supports this conclusion. The smaller variance and standard deviation in the 5-frame interval indicate that measurements are relatively concentrated and stable, whereas the larger variance and standard deviation in the 1-frame and 3-frame intervals imply more dispersed measurements that may be more susceptible to external factors. Based on the analysis of Figs. 14 and 15, it can be inferred that selecting a longer measurement interval (such as 5-frame interval) can improve measurement accuracy to a certain extent in practical applications.

### Comparison of multi-target speed measurement methods

In a normal road environment, the number of detection targets is no longer controlled compared to the previous section, to test whether multi-target detection conditions affect algorithm performance. The method proposed in this paper was compared with the methods by Md.Golam Moazzam et al.^28^, Héctor Rodríguez-Rangel et al.^29^, and D.Bell et al.^15^ through multiple repeated experiments for a comprehensive performance comparison. The comparison results include the minimum accuracy, average accuracy, Multiple Object Tracking Accuracy (MOTA), Multiple Object Tracking Precision (MOTP), Identification F1 score (IDF1), Identification Precision (IDP), Identification Recall (IDR), and frame rate (FPS) of each method. Table 7 presents the comparison results:

**Table 7:** Comparative results of speed estimation accuracy across different methods.

Method	Minimum accuracy	Average accuracy	MOTA	MOTP	IDF1	IDP	IDR	FPS
Héctor	75.7%	86.1%	75.4%	76.4%	86.8%	87.9%	85.9%	10.8
Md.Golam	78.5%	93.7%	74.8%	78.3%	81.8%	82.0%	81.6%	14.3
D. Bell	26.3%	91.9%	76.9%	72.6%	74.6%	77.8%	71.6%	9.5
This work	95.1%	97.6%	80.7%	82.9%	90.1%	91.0%	89.3%	9.8

The results indicate that each approach has its unique advantages and limitations. Héctor Rodríguez-Rangel et al.'s method calculates vehicle speed by drawing the vehicle’s trajectory and determining the speed based on the positions of the start and end points, using YOLOv3 as the target detection algorithm. This method performs well in detection speed, achieving an FPS of 10.8. Although it excels in multi-target tracking results, experiments show that the system’s speed measurement accuracy significantly drops when the vehicle is not moving in a straight line, with the minimum speed measurement accuracy being only 75.7%. This can affect the reliability of speed measurement results in practical applications. Md. Golam Moazzam et al.'s tracking algorithm combines the principles of the three-frame difference method, showing certain advantages in detection speed and demonstrating good target tracking capability. However, in practical applications, this method is easily affected by other moving objects in the video, leading to insufficient robustness in complex scenes. D. Bell et al.'s approach uses a method similar to interval speed measurement, pre-setting two lines in the video. The vehicle is timed from when it crosses the first preset line until it crosses the second preset line. This algorithm employs YOLOv2 + SORT for multi-target tracking tasks. While the multi-target tracking results are relatively lower compared to other algorithms, its simple network structure results in faster recognition speed. This method has high speed measurement accuracy but also faces issues in accurately measuring the vehicle’s real-time speed, especially when the vehicle moves non-linearly between the lines, leading to poor measurement performance.

The method used in this study effectively addresses the common issue in monocular vision speed measurement, where changes in the movement state significantly affect speed measurement accuracy. Although the proposed method has relatively lower speed, the FPS can still be maintained at around 9.8. If a 1/3 or 1/5 frame sampling rate is used, the method can still achieve rapid speed measurement. Additionally, with a computation speed of 9 FPS, the speed measurement interval is only 0.2 s, which is significantly faster than the 0.5 s emergency obstacle avoidance reaction time of a driver. Therefore, if applied to vehicle speed measurement, it would not negatively impact driving safety.For multi-object tracking, the method employs YOLOv7 + DeepSORT, providing a stronger network structure that significantly enhances tracking and speed measurement accuracy while ensuring rapid speed measurement. Although there are inevitable issues such as changes in vehicle posture, occlusion of the target, and inaccurate recognition leading to sudden changes in the bounding box size, which can affect the positioning and consequently the speed measurement, the method’s use of the middle point of the lower edge of the bounding box as the positioning point minimizes the impact on the coordinates of the lower edge middle point. Thus, it effectively reduces the influence on speed measurement accuracy and ensures the stability of the proposed speed measurement algorithm.

## Conclusion

This study proposes a method for fast and accurate speed measurement of moving targets using a monocular camera by combining a two-dimensional positioning algorithm based on distance measurement models with a deep learning-based multi-target detection and tracking algorithm (YOLOv7 + DeepSORT). The network model has been optimized to better suit speed measurement scenarios. By establishing a camera imaging model, the method improves upon the camera calibration process and distance measurement model based on two-dimensional positioning, compensating for errors caused by camera nonlinear imaging distortions and defocusing, thereby enhancing positioning accuracy. This method simplifies the problem from three-dimensional to two-dimensional positioning, reducing computational complexity and proposing a verification scheme that does not require additional equipment, significantly enhancing the accuracy and efficiency of experiments, with a minimum speed measurement accuracy of 95.1%. The method operates autonomously once deployed, independent of external environmental factors. Through varying time intervals for video sampling detection, the method chooses longer intervals to further enhance speed measurement accuracy under the premise of rapid measurement. Not only does this method excel in accuracy, but it also significantly reduces the demand on computational resources, achieving an efficient camera-based speed measurement system.

Future work will build on the findings of this research to explore monocular vision-based three-dimensional calibration and positioning to extend the application scope of the positioning model. Additionally, plans are in place to optimize algorithm performance to make it suitable for mobile devices, thereby reducing dependence on fixed monitoring systems and enhancing the flexibility and diversity of data collection.

## Data Availability

The datasets used and analysed during the current study available from the corresponding author on reasonable request.

## Code availability

The project code has been uploaded to Github. (https://github.com/15849447665/Vehicle-Speed-Measurement-Method).
