# Publisher Correction: Vehicle speed measurement method using monocular cameras

**DOI:** 10.1038/s41598-025-05153-3

**Published:** 2025-06-03

**Authors:** Hao Lian, Meian Li, Ting Li, Yongan Zhang, Yanyu Shi, Yikun Fan, Wenqian Yang, Huilin Jiang, Peng Zhou, Haibo Wu

**Affiliations:** 1 Computer and Information Engineering College, Inner Mongolia Agricultural University, Hohhot, 010000 China; 2 Inner Mongolia Autonomous Region Key Laboratory of Big Data Research and Application of Agriculture and Animal Husbandry, Hohhot, China

Correction to: *Scientific Reports* 10.1038/s41598-025-87077-6, published online 22 January 2025

The original version of this Article contained an error in Equation 10 in the Materials and methods section, under the subheading ‘Two-dimensional positioning’.

$$\begin{array}{c}{O}^{{^{\prime}}{^{\prime}}}N=f\cdot \mathit{tan}\alpha \#\end{array}$$

now reads:

$$\begin{array}{c}{O}^{{^{\prime}}{^{\prime}}}N=f\cdot \mathit{tan}\alpha\end{array}$$

The original Article has been corrected.

